# AlzPharm: integration of neurodegeneration data using RDF

**DOI:** 10.1186/1471-2105-8-S3-S4

**Published:** 2007-05-09

**Authors:** Hugo YK Lam, Luis Marenco, Tim Clark, Yong Gao, June Kinoshita, Gordon Shepherd, Perry Miller, Elizabeth Wu, Gwendolyn T Wong, Nian Liu, Chiquito Crasto, Thomas Morse, Susie Stephens, Kei-Hoi Cheung

**Affiliations:** 1 Interdepartmental Program in Computational Biology and Bioinformatics, Yale University, New Haven, CT, USA; 2 Center for Medical Informatics, Yale University, New Haven, CT, USA; 3 Department of Anesthesiology, Yale University, New Haven, CT, USA; 4 Department of Neurobiology, Yale University, New Haven, CT, USA; 5 Department of Molecular, Cellular and Developmental Biology, Yale University, New Haven, CT, USA; 6 Department of Genetics, Yale University, New Haven, CT, USA; 7 Department of Computer Science, Yale University, New Haven, CT, USA; 8 Initiative in Innovative Computing, Harvard University, Cambridge, MA, USA; 9 Massachusetts General Hospital, Boston, MA, USA; 10 Alzheimer Research Forum; 11 Oracle, Burlington, MA, USA

## Abstract

**Background:**

Neuroscientists often need to access a wide range of data sets distributed over the Internet. These data sets, however, are typically neither integrated nor interoperable, resulting in a barrier to answering complex neuroscience research questions. Domain ontologies can enable the querying heterogeneous data sets, but they are not sufficient for neuroscience since the data of interest commonly span multiple research domains. To this end, e-Neuroscience seeks to provide an integrated platform for neuroscientists to discover new knowledge through seamless integration of the very diverse types of neuroscience data. Here we present a Semantic Web approach to building this e-Neuroscience framework by using the Resource Description Framework (RDF) and its vocabulary description language, RDF Schema (RDFS), as a standard data model to facilitate both representation and integration of the data.

**Results:**

We have constructed a pilot ontology for BrainPharm (a subset of SenseLab) using RDFS and then converted a subset of the BrainPharm data into RDF according to the ontological structure. We have also integrated the converted BrainPharm data with existing RDF hypothesis and publication data from a pilot version of SWAN (Semantic Web Applications in Neuromedicine). Our implementation uses the RDF Data Model in Oracle Database 10g release 2 for data integration, query, and inference, while our Web interface allows users to query the data and retrieve the results in a convenient fashion.

**Conclusion:**

Accessing and integrating biomedical data which cuts across multiple disciplines will be increasingly indispensable and beneficial to neuroscience researchers. The Semantic Web approach we undertook has demonstrated a promising way to semantically integrate data sets created independently. It also shows how advanced queries and inferences can be performed over the integrated data, which are hard to achieve using traditional data integration approaches. Our pilot results suggest that our Semantic Web approach is suitable for realizing e-Neuroscience and generic enough to be applied in other biomedical fields.

## Background

e-Science involves developing tools, technologies, and infrastructure to support multidisciplinary and collaborative science enabled by the Internet [1]. One of the challenges that e-Science aims to address is data integration. e-Neuroscience [2], otherwise known as neuroinformatics, shares the same vision as e-Science but focuses on the neurosciences. It is also encompassed by the informatics-oriented goal of the Human Brain Project, which emphasizes the importance of integrating heterogeneous neuroscience-related information from the molecular level to the behavioral level [3]. Integrating neuroscience data, including sequence data, molecular data, disease data and behavioral data, will be a significant step towards better understanding brain function [4].

By combining the experimental results produced by multi-disciplinary groups, one can allow a more thorough investigation and understanding of complex neuroscience research problems, including the study of neurodegenerative diseases such as Alzheimer's Disease (AD) and Parkinson's Disease (PD) [2]. Below, we discuss some of the challenges involved in integrating rapidly growing heterogeneous and distributed neuroscience data.

**Figure 1:**
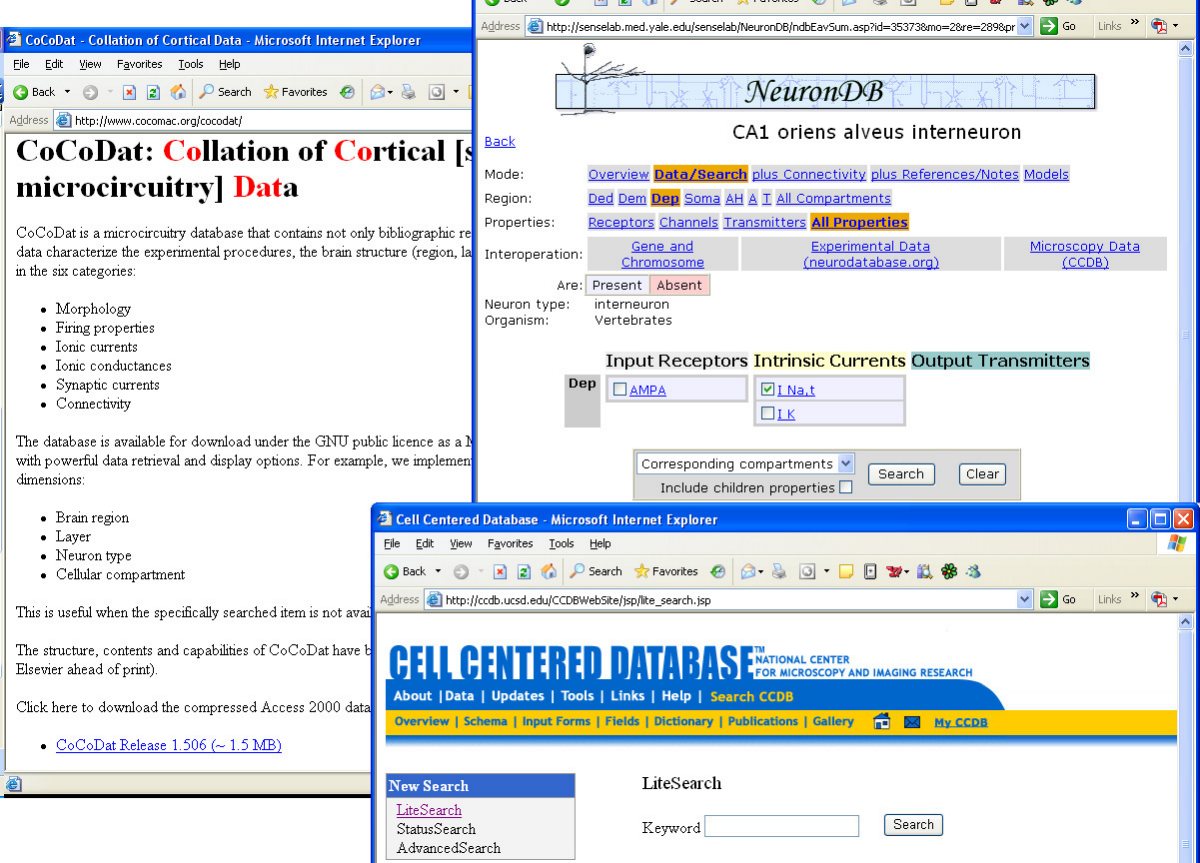
**Examples of three neuroscience database Web interfaces**. The Web interfaces of CocoDat, NeuronDB, and the Cell Centered Database.

1. **Registry**. A large number of neuroscience resources have been developed independently to address various research needs. While search engines (e.g., Google) can help users locate neuroscience resources of interest, such keyword based search approaches suffers from the problem of specificity and sensitivity. For example, if a search is performed using the keyword "neuron", a large number of hits will be returned. To address this problem, central registries of neuroscience resources have been created to categorize and keep track of existing neuroscience data sets. These registries provide search interfaces for users to find data of potential interest. The Neuroscience Database Gateway (NDG) [5] is one such example. NDG was launched in 2004 as a pilot project sponsored by the Society for Neuroscience, with an exclusive focus on categorizing neuroscience resources. It employs a set of standard terms (e.g., name, description, URL, and species) for describing each resource (e.g., a database or a software tool). As the number of neuroscience resources continues to grow, such a centralized approach to registering resources may not be easily maintainable (it is difficult for a single person or a single group to keep track of such a rapidly growing collection of resources). A better and more efficient framework that allows registration and discovery of this kind of distributed resource will be necessary.

2. **Interface**. Within NDG each of the different data sources has its own data format and interface. For example, Cell-Centered Database (CCDB) [6] (cellular imaging data) provides a free text search interface; SenseLab [7] (integration of multidisciplinary sensory data) has a structured form search interface; and CoCoDat [5] (cortical cell and micro-circuitry data) is available for download as a Microsoft Access database. Examples of the differing Web interfaces are shown in Figure 1. Although each of these data sources contains different types of data, they refer to common bio-entities. Such heterogeneity in data format and user interface makes data interoperability and data analysis difficult, yet currently the only way to integrate the data is to do it manually. A standardized and machine-understandable data format with an open and unified data access model is crucial to building a data integration framework for e-Neuroscience.

3. **Nomenclature**. One of the difficulties in enabling neuroscience data sources to be broadly sharable is a lack of standard nomenclature. For example, different terms (e.g., Neural Arch and Vertebral Arch) may be used to describe the same neuro-anatomical region (e.g., part of the spinal cord). Ambiguity also arises when the same term is associated with multiple meanings (e.g., spine could mean vertebral spine or dendritic spine). It would be highly advantageous if there were an e-Neuroscience framework that could better reconcile the ambiguities.

4. **Granularity**. Different neuroscience data sources may model the same type of data at different levels of granularity. For example, CCDB uses a single "dendrite" compartment for all data associated with dendrites, whereas NeuronDB (a subdatabase of SenseLab) subdivides dendrites into types (e.g., apical and basal) and compartments (e.g., proximal, medial, and distal). As a result, data within NeuronDB can be associated with specific dendritic compartments, which is not possible in CCDB. An ideal data framework would be able to model data at differing levels of granularity.

### Semantic Web approach to representing and integrating data

The primary goal of the Semantic Web is to expose the semantics of Web-accessible data using a machine-readable knowledge representation format so that data can be more easily interpreted and integrated by computer programs (or Web agents). As a result, the Semantic Web consists of components that aim to fulfill the requirements in this realm. The fundamental components of the Semantic Web include the following: knowledge representation, ontological languages, and Semantic-Web-aware tools.

### Knowledge representation

Knowledge representation comes in different forms that exhibit different levels of complexity. A **controlled vocabulary **is a knowledge base that holds definitions of terms. A **thesaurus **is a more expressive knowledge base that, in addition, holds assertions regarding the semantic relationships between the terms. An **ontology **is a specification of a representational vocabulary for a shared domain of discourse [8]. It captures domain concepts and their relations and properties. Ontologies can be categorized into upper level ontology (contains common and generic knowledge that can be shared across different domains), middle level ontology (contains focused domain spanning knowledge), and domain level ontology (contains domain-specific knowledge) [9]. With the application of the Semantic Web, controlled vocabularies, thesauri, and ontologies are exposed to processing by Web-aware agents, as well as to human access and interpretation. This facilitates extensible knowledge representation and semantic interoperability, and critically deepens our ability to treat the Web as a true knowledge base.

Recognizing the increasing need for using expressive bio-ontologies to facilitate machine-based data integration and inference, community efforts have begun to build ontologies for use by computer applications deployed in different domains of biosciences. Examples include the Gene Ontology [10] (a controlled vocabulary describing gene and gene product attributes), Plant Ontology [11] (a controlled vocabulary describing plant structures, growth, and developmental stages), and Unified Medical Language System [12] (a vocabulary database about biomedical and health related concepts). In response to the growing number of bio-ontologies, the National Center for Biomedical Ontologies (NCBO) [13] was established to enable researchers to find, create, disseminate, and manage biomedical information and knowledge in a machine-processable form. The Center's resources include the Open Biomedical Ontologies library [14], the Open Biomedical Data (OBD) repositories, and tools for accessing and using these biomedical ontologies and their associated data in research. Many of the ontologies hosted by the NCBO can be cross-referenced or inter-linked to facilitate more comprehensive knowledge acquisition, although much research is still in progress to help determine equivalence across ontologies, and to further explore automating the labor intensive mapping process. There are also ongoing efforts to create upper level ontologies for disparate domains. This kind of ontology focuses on providing a set of general concepts upon which domain-specific ontologies (e.g., microarrays, proteomics, and pathways) could be constructed. Examples in the biological domain include the Functional Genomics Investigation Ontology (FuGO) [15], and Ontology of EXPeriment (EXPO) [16].

In research investigations that commonly span domains, such as neuroscience, providing the ability to construct upper ontologies and bridge ontologies is critical to interoperability.

### Semantic Web languages

To enable computers to process, understand, and inference over an ontology, it is necessary to have a computer language, or what we call an ontological language, to formalize an ontology in a way that it can be reasoned over by software automatically. It is also essential to have a common format that can facilitate the interchange of data. To this end, the W3C has recommended two standards for building an ontology in the Semantic Web – Resource Description Framework (RDF) [17] and Web Ontology Language (OWL) [18].

RDF models concepts and their instances in a format called a triple. A triple is an RDF statement which contains a subject, a predicate and an object about a resource where the subject is the resource itself, the predicate is the relationship between the resource and the object, and the object can be another resource or a data value. RDF in fact can be specified in different syntax formats, although the most commonly used format is the RDF/XML, which employs the eXtensible Markup Language (XML) to structure its representation of resources. Descriptions of the concepts and their relations (e.g., subclass/superclass) are specified separately in a specialized RDF format called RDF Schema (RDFS) [19]. The following example illustrates an RDF statement:

<,

,

>

expressing that *Dopamine *has the *Function *of being a *Neurotransmitter*. Each component of the triple is identified using a Uniform Resource Identifier (URI) [20]. When resources have the same URI they are assumed to be the same entity, and any data about the entity can be merged. As triple statements become connected together, they form a directed labeled graph.

OWL extends RDF by adding more vocabulary to describe the relations such as cardinality and equality among classes and properties. Advanced knowledge representation includes making assertions or claims about explicit objects (e.g., "acetylcholinesterase is an enzyme"). Representing knowledge in such an explicit form in OWL is based on Description Logics, which enables computers to draw new conclusions from existing knowledge. Insights from the Description Logics research community have had a strong influence on the design of OWL, particularly on the formalization of the semantics, the choice of language constructors, and the integration of data types and data values [21].

We have chosen to use RDFS for knowledge representation in the initial stages of this project, as it is well developed, widely used, and expressive enough for our case.

### Semantic Web-aware tools

Ontologies (written in RDFS or OWL) are the key components of the Semantic Web. Without suitable tools for developing, processing, storeing, and inferencing over the data, it would not be possible to infer new knowledge could hardly be inferred. Consequently, a large number of open source and commercial Semantic Web tools have been developed. They are:

1. **Ontology editors and visualization tools**. These tools allow users to develop, edit, and visualize ontologies and their associated data. Examples include Protégé [22], WebOnto [23], and GrOWL [24]. There are also advanced ontology editors that allow alignment and integration of multiple ontologies (e.g., COBrA [25]).

2. **Parsers**. To enable the development of computer applications that utilize and process ontologies, RDF and OWL parsers have been made available for most popular programming languages. For example, PerlRDF is one of the RDF parsers written in Perl [26]. Jena is a framework for building Semantic Web applications and for parsing RDF, RDFS and OWL in the Java programming environment [27].

3. **Database and querying tools**. To provide persistence, management and querying capabilities for RDF/OWL, several RDF database systems have been implemented. Among them, Sesame (a.k.a OpenRDF) [28] and Kowari [29] are open-source RDF database systems while the Oracle RDF Data Model [30] is a feature of the Oracle Database and therefore a commercial offering. Some of these database systems (e.g., Sesame) implement their RDF query languages in compliance with the SPARQL standard specifications [31]. Besides, tools such as D2RQ [32] are also available for mapping relational schema to OWL/RDFS ontologies.

4. **Reasoners**. To support reasoning based on Description Logics specified in OWL, a number of reasoners have been developed including Racer [33], FaCT [34], and Pellet [35].

## Results

**Figure 2:**
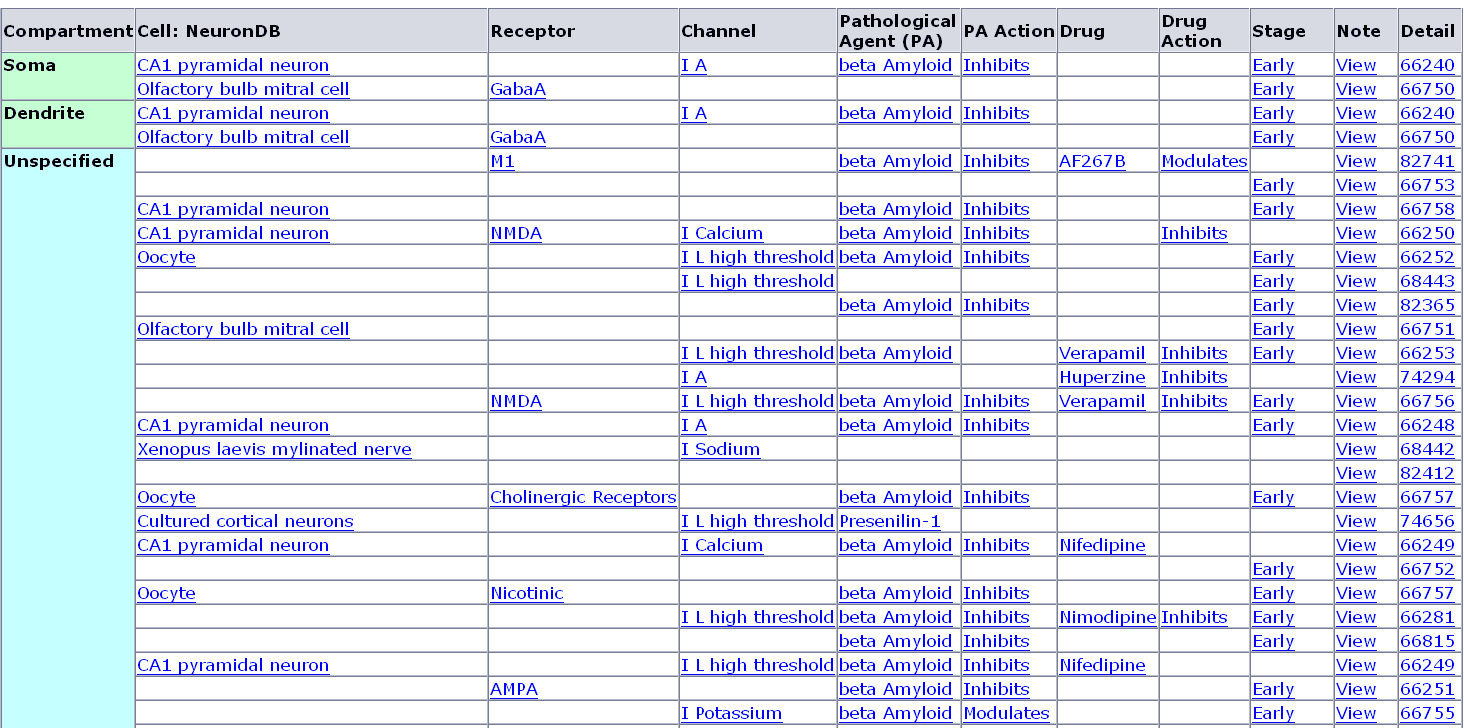
**Partial BrainPharm data**. A portion of the pilot data set retrieved from the BrainPharm database.

As described in the Methods section, we hand-crafted an ontology for BrainPharm using RDFS. We then instantiated the ontology with some pilot data, which our neuroscientists extracted from a subset of the BrainPharm data. Figure 2 shows a portion of the pilot data set (pathological mechanisms) in tabular format. The first four columns contain information about different types of neurons including their neuronal properties, such as receptors and channels localized to different neuronal compartments. The remaining columns capture information about (i) the pathological changes caused by certain pathological agents (e.g., beta Amyloid) to the neuronal properties (e.g., beta Amyloid inhibits the I Calcium channel of CA1 pyramidal neuron), (ii) the drugs and their actions on the pathological changes (e.g., Nifedipine reduces the pathological effect of beta Amlyloid on the molecular properties of CA1 pyramidal neuron), (iii) stages of the disease (e.g., early, middle, and late stages), and (iv) literature sources (e.g., PubMed sources).

We loaded a subset of BrainPharm in RDF, and a subset of SWAN in RDF, into the ORACLE RDF Data Model.  We then created inference rules based upon the RDFs. In our pilot use case, we loaded: (i) the BrainPharm drug-related data including the drug property and drug action information related to the pathological mechanisms underlying AD, and (ii) the SWAN data including publication, hypothesis, and annotation information [36]. This approach is potentially easier to manage and adapt than integrating many data sets using a relational model, as no schema has to be pre-defined for our RDF models.

As a demonstration, we developed a Web-based application called "AlzPharm" [37] which allows users to relate the drug information from BrainPharm to the publication information stored in SWAN. Our Web interface uses Java server faces to render different information into different User Interface (UI) components, and the connection to the Oracle database is made available by Java Database Connectivity (JDBC).

**Figure 3:**
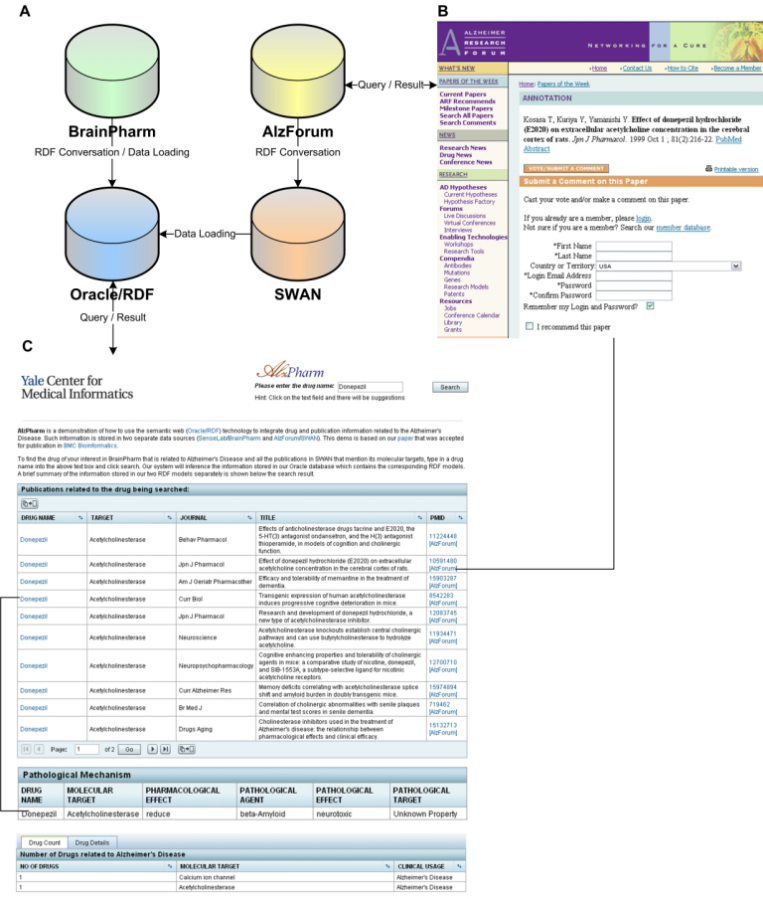
**The AlzPharm Web application**. (A) The data sources of the application. (B) The AlzForum website. (C) The interface of the application.

Figure 3 shows some of the UI components of our demo and depicts a schematic data flow of the information transferring from their original data sources to the Oracle RDF Data Models. Figure 3A shows that the data originating from BrainPharm are loaded into our database directly after RDF conversion, and that the data originating from AlzForum [38] are converted into RDF, made available by SWAN at their website, and then loaded into our database. Figure 3B shows the original Web interface to the AlzForum data repository, which is not in RDF format. Figure 3C shows part of the Web query interface that we developed to allow users to query data across both the BrainPharm and SWAN datasets.

### An integrated query

Our Web interface provides not only information about the individual datasets in our database, but also a simple text field for scientists to enter a drug name for finding the publications in SWAN that mention the molecular targets of interest. The drug being searched has to exist in the BrainPharm dataset, otherwise there will be no result. For this reason, we also provide drug name suggestions, enabled by the Asynchronous JavaScript and XML (AJAX) technology, to the users based the data in real time. After our system receives the search request from the user, it executes the following SQL query statement and queries the underlying RDF models with the specified drug name:

SELECT distinct drugname DRUG_NAME, target TARGET,

      journal JOURNAL, title TITLE, pmid PMID

FROM TABLE(

      SDO_RDF_MATCH(

            '(?drug b:name ?drugname)

            (?drug b:hasMolecularTarget ?target)

            (?mech b:hasPharmacologicalAgent ?drug)

            (?mech b:hasPharmacologicalTarget ?path)

            (?path b:hasPathology ?disease)

            (?disease b:name ?disname)'

            ,

         SDO_RDF_Models('brainpharm'),

         SDO_RDF_Rulebases('RDFS'),

   SDO_RDF_Aliases(SDO_RDF_Alias('b','')),

            'lower(disname) = "alzheimer""s disease"'

      )

   ) bpharm,

   TABLE(

      SDO_RDF_MATCH(

            '(?pub s:title ?title)

            (?pub s:journal ?journal)

            (?pub s:abstract ?abs)

            (?pub s:pmid ?pmid)

            (?pub rdf:type s:Publication)'

            ,

         SDO_RDF_Models('swan'),

         SDO_RDF_Rulebases('RDFS'),

   SDO_RDF_Aliases(SDO_RDF_Alias('s',')),

         null

      )

   ) swan

   where regexp_like(swan.abs, bpharm.target, 'i') and lower(drugname) = lower(?)

The query results shown in Figure 3C list the SWAN publications related to the drug Donepezil (with acetylcholinesterase being the molecular target of the drug), which is indication by "?" at the end of the query. The user can click on the drug name to get more detailed information directly from BrainPharm about the effect of the drug on some known pathological mechanism(s) related to AD. In addition, users can also click on the AlzForum link under the PMID (PubMed ID) column to go to AlzForum for additional comments that have been given by AD researchers for that publication, as shown in Figure 3B.

The results demonstrated how a complex query can be formulated to integrate BrainPharm's drug data and SWAN's publication data. In addition, it also demonstrated the use of RDF inferencing based on the parent-child (*is-a*) relationship between the *Publication *class (e.g., original articles retrieved from PubMed) and *ARFPublication *class (e.g., PubMed articles that have been commented by researchers/curators associated with AlzForum) as defined in the SWAN RDF Schema and shown below.

<rdf:Description rdf:about="ARFPublication">

   <rdfs:label>ARFPublication</rdfs:label>

   <rdf:type rdf:resource="Class"/>

   <rdfs:subClassOf rdf:resource="Publication"/>

</rdf:Description>

Since our query has specified retrieval of all the related *Publication*s (?pub rdf:type s:Publication) from the dataset, the Oracle RDF Data Model will identify all the publications – including the ARF publications, which are related to AD drugs (e.g., Donepezil) based on the RDFS rules that contain their relationship we defined. Although the hierarchical relation here only has two levels, the *is-a *inference could be applied to any number of levels. Semantic inferencing is not directly supported by the relational approach.

### A "group-by" query

As shown in Figure 3C (bottom), we queried BrainPharm to group and count AD drugs based on their molecular targets and clinical usage. The SQL query statement is as follows:

SELECT count(distinct bpharm.drugname) NO_OF_DRUGS,

   bpharm.target MOLECULAR_TARGET, bpharm.disname CLINICAL_USAGE

FROM TABLE(

   SDO_RDF_MATCH(

         '(?drug b:hasMolecularTarget ?target)

         (?mech b:hasPharmacologicalAgent ?drug)

         (?mech b:hasPharmacologicalTarget ?path)

         (?path b:hasPathology ?disease)

         (?drug b:name ?drugname)

         (?disease b:name ?disname)'

         ,

      SDO_RDF_Models('brainpharm'),

      SDO_RDF_Rulebases('RDFS'),

   SDO_RDF_Aliases(SDO_RDF_Alias('b','')),

         'lower(disname) = "alzheimer""s disease"'

   )

) bpharm

group by bpharm.target, bpharm.disname

The output of this query indicates that there are two groups of drugs available for AD. The first one contains one drug, which molecular target is acetylcholinesterase. The second group also contains one drug but its molecular target is calcium ion channel. The query demonstrated how to make use of the "GROUP BY" feature (which is supported by standard SQL) to perform aggregation on RDF data. Implementations of other RDF query languages by other RDF databases do not support aggregate functions such as "COUNT", "SUM" and "AVERAGE" with "GROUP BY". The Oracle Database has the advantage of the RDF query being embedded within a SQL statement.

## Conclusions and future directions

As Sir Tim Berners-Lee has reinforced, today most of the world's data are still locked in large data stores and are not published as an open Web of inter-referring resources [39]. Areas such as neuroscience, which rely heavily on analyzing a tremendous amount of data of disparate and diverse types, cannot fully leverage the potential of the available knowledge that is captured in this way. There is an emerging need for an infrastructure that can facilitate the interchange of such data. In this paper, we have shown the benefits of exposing data in RDF format, which can be shared, integrated, and reasoned about. We have also shown how to use the Oracle RDF Data Model to create a Semantic Web repository for integrating data relating to AD from BrainPharm and SWAN. We further demonstrated the RDF querying and RDFS inferencing features, including the support of data aggregation functions (based on traditional SQL) and semantic inference rules (based on RDFS) provided by the Oracle RDF Data Model, which can hardly be achieved by traditional data integration. The Oracle Database's extensions to SQL for querying RDF data are particularly powerful – allowing relational data to be queried alongside RDF data. For example, one can formulate a complex nested query that retrieves data from both an RDF graph and a relational table and join the query results using a relational join. Technically, our approach can also be adapted to other integration solutions such as data warehousing and query mediation.

While neuroscientists always need to access and integrate biomedical data that span multiple disciplines, integrating neuroscience data using our proposed Semantic Web approach appears to be effective, based on our results. We believe that our approach is the robust candidate for e-Neuroscience and could be generalizable to be applied in other biomedical fields.

To increase the use of Semantic Web in e-Neuroscience, we suggest the following future work:

1. **User-friendly query interface**. We will extend the Web-based application to allow users to perform more kinds of queries (e.g., queries that are based on drug properties and neuronal properties).

2. **Enhanced integration**. To support better integrative neuroscience research, we will strengthen the linkage between BrainPharm and SWAN. While we are in the process of enhancing the ontological representation of BrainPharm and SWAN, more AD-related data are being added to the two databases.

3. **OWL support**. Oracle Database 10g release 2 provides support for storing, querying, and inferencing over RDF and RDFS. Currently, it is also possible to store OWL in the Oracle RDF Data Model, but OWL inferencing can only be performed indirectly through application layer functionality. The next release of the Oracle Database will provide native support for OWL and we plan to take advantage of this capability to better integrating disparate data sources and ensure more advanced inferencing.

4. **Query mediation**. The data integration system we demonstrated focuses on building a central repository of data. We are interested in exploring a federated data approach, where the query is mediated across distributed data sources. Efforts in this area are ongoing within the computer science research community (e.g., [40]). Initial work has started within the life science domain, e.g., Stephens et al. have described a federated database approach for querying drug safety data [41].

5. **Use case**. To make Semantic Web technologies useful to neuroscience researchers, it is important to drive our Semantic Web development by real use cases. While SenseLab focuses on data at the molecular and basic research level, AlzForum focuses on cataloging and documenting research hypotheses (including clinical trial studies) relating to AD. The potential benefit of integrating SenseLab and AlzForum is to support translational research in AD. We will develop use cases in this translational research context. For this, we will need to interact closely with domain experts.

## Methods

We used the Oracle RDF Data Model provided by Oracle Database10 g release 2 to store and semantically integrate data from two independently created neuroscience data sources, namely, BrainPharm (a subset of SenseLab [7]) and SWAN [36]:

### Data sources

#### BrainPharm

**Figure 4:**
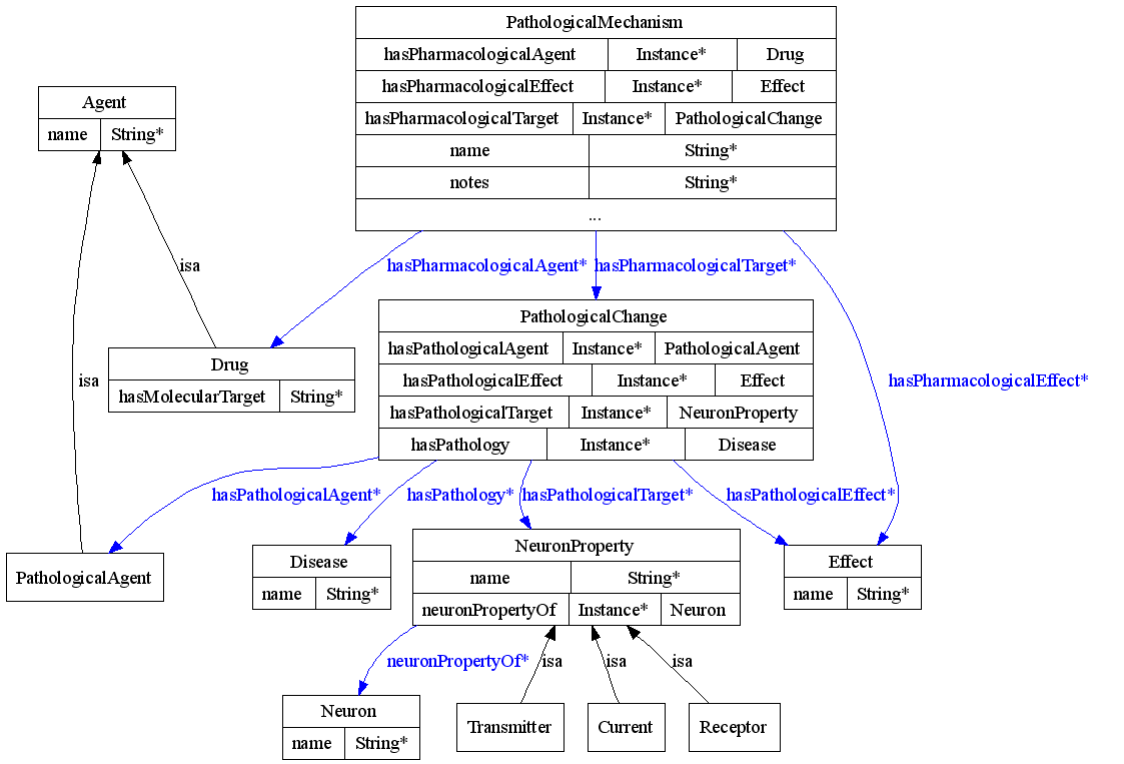
**The BrainPharm ontology**. A simplified schematic representation of the ontology for the BrainPharm data.

BrainPharm includes data for support of research on drugs for the treatment of neurological disorders [42]. It contains information about the effect of drugs on pathological (or molecular) mechanisms (which involve neuronal properties such as receptors, currents, and neurotransmitters) mediating the pathological changes in various neurological disorders such as AD. Figure 4 shows the ontology diagram of BrainPharm, which was created manually using Protégé. As shown in the diagram, the main classes include: *disease *(e.g., AD), *drug *(subclass of *agent*), *pathological mechanism *(which contains related pharmacological and pathological information), *pathological change *(which has a *pathological agent *and its *effect *and *targets*), *neuron *(e.g., CA1 pyramidal neuron), and *neuron property *(which has the following subclasses: *transmitter*, *current*, and *receptor*). There are also non-hierarchical relationships among these classes. For example, *pathological mechanism *relates to *drug *through the *hasPharmacologicalAgent *property. The BrainPharm ontology was designed and populated based on the input from our neuroscientists.

#### SWAN/AlzForum

**Figure 5:**
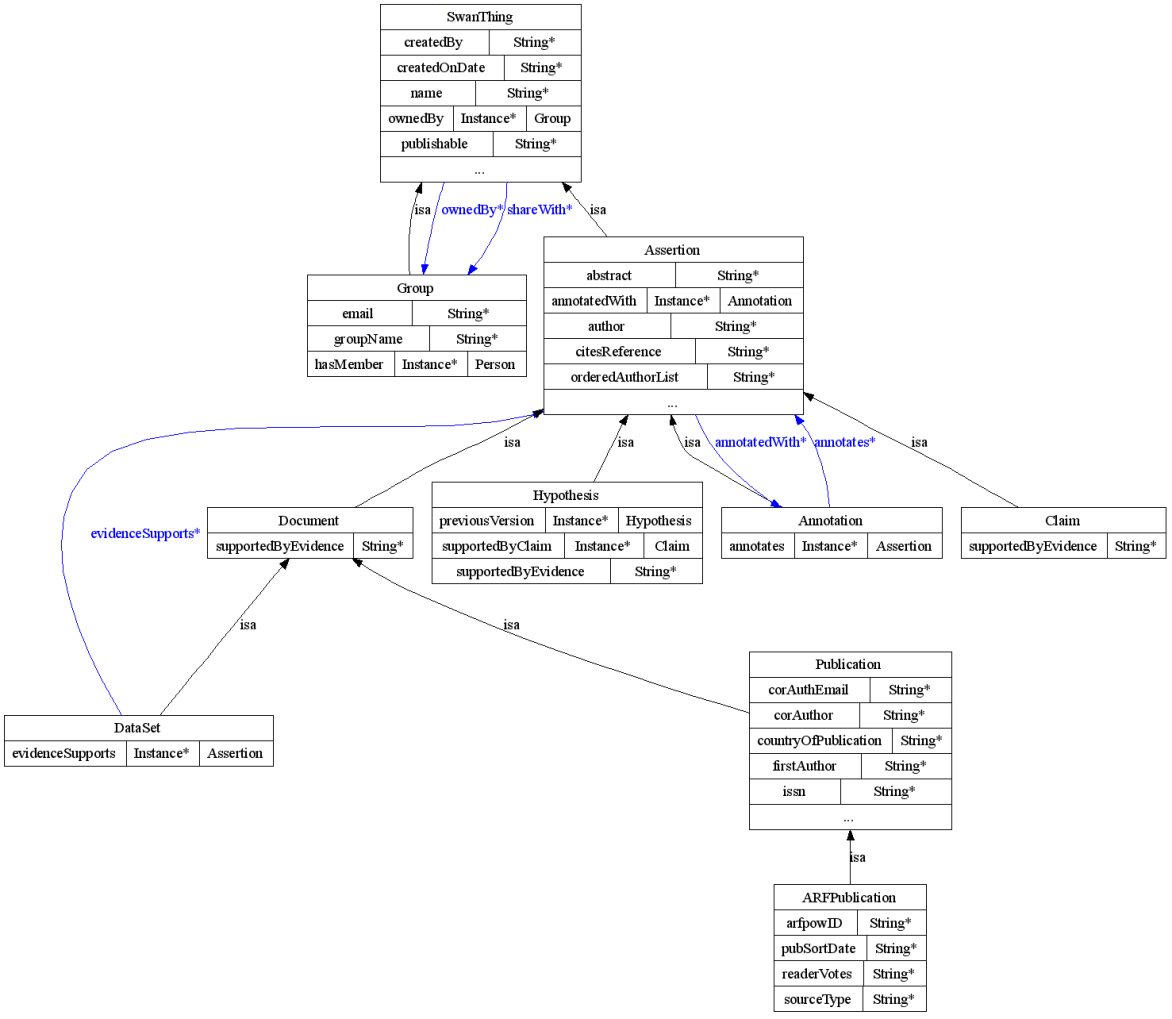
**The SWAN ontology**. A simplified schematic representation of the ontology for the SWAN data.

SWAN is a project to develop knowledge management tools and resources for AD researchers, based on an ecosystem model of scientific discourse [43]. The SWAN project is currently developing an OWL ontology for representing information about scientists, experiments, publications, scientific data, bibliographic data, scientific ontologies, biomedical research collaborations, and scientific Web communities. A beta release of SWAN is now under development, with planned deployment on the Alzheimer Research Forum Web site [44] in early to mid 2007. AlzForum [38] is a widely used scientific Web community, which reports on the latest Alzheimer's scientific research, and develops data sources of genes, scientific articles, animal models, antibodies, medications, grants, research jobs, and more. Prior to employing OWL to implement the Semantic Web, a SWAN pilot version was implemented in 2005–2006 using RDF/S to represent the data (we acquired in this project). Figure 5 shows the ontology diagram of a portion of the SWAN pilot knowledgebase.

### Data conversion and storing

As a pilot demonstration, we have integrated the drug-related information extracted from BrainPharm that is related to AD with the SWAN hypothesis and publication information extracted from SWAN/AlzForum. We have manually created the RDFS for BrainPharm as described before and converted the extracted data into RDF. Since the SWAN data are already available in RDF format, we then loaded both the BrainPharm and SWAN data, including their corresponding RDFS, into the Oracle RDF Data Model using its data loader tool, which supports loading RDF in N-triple format. As a result, we used Jena to simply convert the RDF/XML into N-triple before we loaded the data. While SWAN already has its own namespace for URIs, we defined our BrainPharm namespace for URIs so that data values referenced by different URI's could be differentiated and joined correctly.

### RDF queries

We used the SPARQL-like RDF query syntax required by the Oracle RDF Data Model to query our data in RDF. Examples of such kind of queries are illustrated in the results section.

### Web application

Our Web application has been implemented using the Java Web technology. We have also used AJAX on the Web page to perform asynchronous query to the server so as to provide some non-critical information, such as drug name suggestion in the search, in a timely and non-interruptive manner. Moreover, we have used Java Server Faces to render different information, such as drug count and search result, into different UI components on the interface. Our application has been deployed to a Tomcat Web Application Server 5.5 running on a SUSE Linux machine with four Intel Xeon CPUs at 2.80 GHz and 4 GB memory, which is where the Oracle Database is also running.

## Competing interests

The authors declare that they have no competing interests.

## Authors' contributions

HL implemented AlzPharm using the Oracle RDF Data Model and built the Web user interface. KC is responsible for the Semantic Web development of SenseLab. GS, PM, CC, NL, TM, and KC are members of the SenseLab/BrainPharm team. TC, YG, EW, JK, and GW are members of the AlzForum/SWAN team. SS provided technical help on the Oracle RDF Data Model. All authors have contributed to the final version of the manuscript.
